# Probing deformed commutators with macroscopic harmonic oscillators

**DOI:** 10.1038/ncomms8503

**Published:** 2015-06-19

**Authors:** Mateusz Bawaj, Ciro Biancofiore, Michele Bonaldi, Federica Bonfigli, Antonio Borrielli, Giovanni Di Giuseppe, Lorenzo Marconi, Francesco Marino, Riccardo Natali, Antonio Pontin, Giovanni A. Prodi, Enrico Serra, David Vitali, Francesco Marin

**Affiliations:** 1 Physics Division, School of Science and Technology, University of Camerino, via Madonna delle Carceri 9, Camerino (MC) I-62032, Italy; 2 INFN, Sezione di Perugia, Via A. Pascoli, Perugia I-06123, Italy; 3 Nanoscience-Trento-FBK Division, Institute of Materials for Electronics and Magnetism, Povo (TN) I-38123, Italy; 4 Istituto Nazionale di Fisica Nucleare (INFN), Trento Institute for Fundamental Physics and Application, Povo (TN) I-38123, Italy; 5 Dipartimento di Fisica e Astronomia, Università di Firenze, Via Sansone 1, Sesto Fiorentino (FI) I-50019, Italy; 6 INFN, Sezione di Firenze, Via Sansone 1, Sesto Fiorentino (FI) I-50019, Italy; 7 CNR-Istituto Nazionale di Ottica, Largo E. Fermi 6, Firenze I-50125, Italy; 8 Dipartimento di Fisica, Università di Trento, Povo (TN) I-38123, Italy; 9 Centre for Materials and Microsystem, Fondazione Bruno Kessler, Povo (TN) I-38123, Italy; 10 Department of Microelectronics and Computer/ECTM/DIMES Technology Centre, Delft University of Technology, Feldmanweg 17, 2628 CT Delft, PO Box 5053, Delft 2600 GB, The Netherlands; 11 European Laboratory for Non-Linear Spectroscopy (LENS), Via Carrara 1, Sesto Fiorentino (FI) I-50019, Italy

## Abstract

A minimal observable length is a common feature of theories that aim to merge quantum physics and gravity. Quantum mechanically, this concept is associated with a nonzero minimal uncertainty in position measurements, which is encoded in deformed commutation relations. In spite of increasing theoretical interest, the subject suffers from the complete lack of dedicated experiments and bounds to the deformation parameters have just been extrapolated from indirect measurements. As recently proposed, low-energy mechanical oscillators could allow to reveal the effect of a modified commutator. Here we analyze the free evolution of high-quality factor micro- and nano-oscillators, spanning a wide range of masses around the Planck mass *m*_P_ (≈22 μg). The direct check against a model of deformed dynamics substantially lowers the previous limits on the parameters quantifying the commutator deformation.

## Abstract (web summary)

A minimal observable length leading to deformed commutation relations is a common feature of theories that aim to merge quantum physics and gravity. Here, the authors analyse the free evolution of micro- and nano-oscillators to lower the limits on the parameters quantifying commutator deformation.

## Graphical abstract

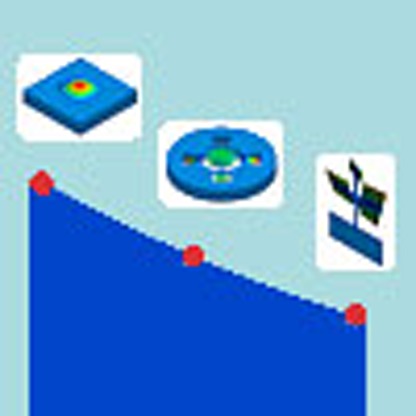

The emergence of a minimal observable length, at least as small as the Planck length 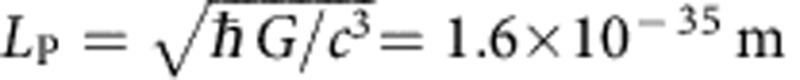, is a general feature of different quantum gravity models^1^,^2^. In the framework of quantum mechanics, the measurement accuracy is at the heart of the Heisenberg relations, that, however, does not imply an absolute minimum uncertainty in the position. An arbitrarily precise measurement of the position of a particle is indeed possible at the cost of our knowledge about its momentum. This consideration motivated the introduction of generalized uncertainty principles (GUPs)^1^,^2^,^3^,^4^,^5^,^6^,^7^,^8^, such as


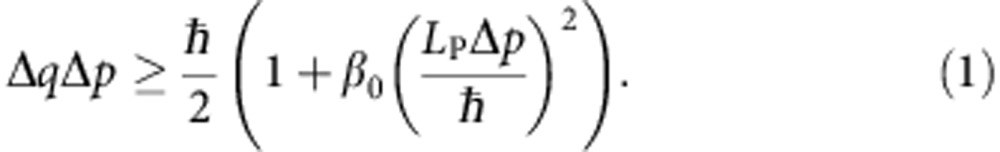


Equation 1 implies indeed a nonzero minimal uncertainty 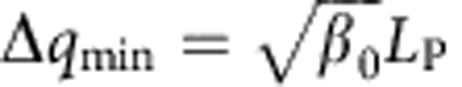. The dimensionless parameter *β*_0_ is usually assumed to be around unity, in which case the corrections are negligible unless energies (lengths) are close to the Planck energy (length). However, since there are no theories supporting this assumption, the deformation parameter has necessarily to be bound by the experiments. Any experimental upper limit for *β*_0_>1 would constrain new physics below the length scale 
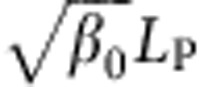
9.

A direct consequence of relation (1) is an increase of the ground state energy *E*_min_ of an harmonic oscillator. Recently, an upper limit to *E*_min_ has been placed by analysing the residual motion of the first longitudinal mode of the bar detector of gravitational waves AURIGA^10^,^11^. Although the imposed bound, *β*_0_<10^33^, is extremely far from the Planck scale, it provides a first measurement just below the electroweak scale (corresponding to 10^17^ *L*_P_).

To the GUP (1) it is possible to associate a modified canonical commutator^1^,^3^,^4^:


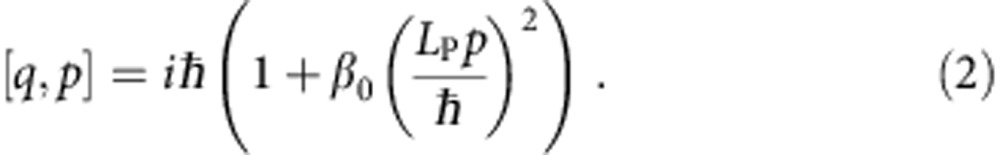


Its introduction represents a further conceptual step, as it defines the algebraic structure from which the GUP should follow, and it implies changes in the whole energy spectrum of quantum systems, as well as in the time evolution of a given observable.

Because of its importance as a prototype system, several studies have been focused on harmonic oscillators. Modifications of stationary states are calculated in refs 12, 13, 14. Approaches to construct generalized coherent states are proposed in refs 15, 16. The modified time evolution and expectation values of position and momentum operators are discussed in refs 17, 18, 19, whereas in ref. 20 Chen *et al.* calculated the temporal behaviour of the position and momentum uncertainties in a coherent state, finding a squeezing effect.

In spite of this huge theoretical interest, the subject suffers from the complete lack of dedicated experiments and so far limits to the deformation parameters have been extrapolated from indirect measurements^9^,^21^,^22^. It has recently been proposed that the effect of a modified commutator could be revealed by studying the opto-mechanical interaction of macroscopic mechanical oscillators^23^. Here we elaborate a different experimental protocol and describe a set of dedicated experiments with state of the art micro- and nano-oscillators. We show that, in the Heisenberg picture of quantum mechanics and assuming the validity of the commutator (2) for the coordinates of the centre-of-mass (c.m.), the time evolution of its position exhibits an additional third harmonic term and a dependence of the oscillation frequency on its amplitude. The strength of such effects depends on *β*_0_. We then analyse the dynamics of different oscillators to place upper bounds to the parameters quantifying the deformation to the standard quantum-mechanical commutator. Such bounds span a wide range of test mass values, around the landmark given by the Planck mass. Previous limits, derived indirectly from the analysis of some metrological experiments, are substantially lowered, by several orders of magnitude.

## Results

### Theoretical model

The basic idea of our analysis is assuming that the commutation relations between the operator *q* describing a measured position in a macroscopic harmonic oscillator, and its conjugate momentum *p*, are modified with respect to their standard form. In other words, and more generally, we suppose that the deformed commutator should be applied to any couple of position/momentum conjugate observables that are treated in a quantum way in experiments and standard theories. At the same time, we keep the validity of the Heisenberg equations for the temporal evolution of an operator 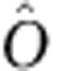, that is, 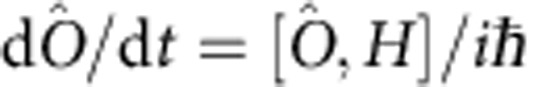, where *H* is the Hamiltonian. For an oscillator with mass *m* and resonance angular frequency *ω*_0_, we also assume that the Hamiltonian maintains its classical form 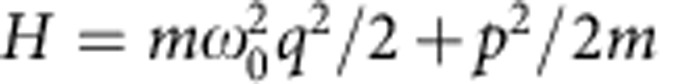. Such hypotheses are also underlying the proposal of ref. 23.

We first define the usual dimensionless coordinates *Q* and *P*, according to 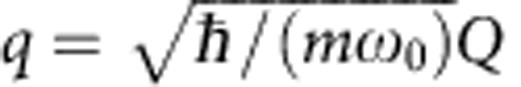 and 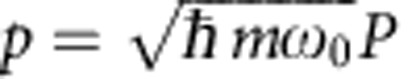. The Hamiltonian is now written in the standard form 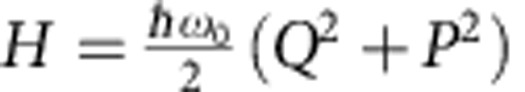 and the commutator of Equation 2 becomes


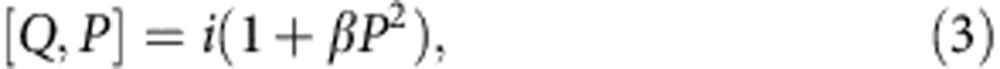


where 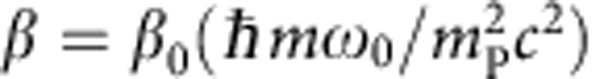 is a further dimensionless parameter that we assume to be small (*β*<<1). Such assumption will have to be consistent with the experimental results. We now apply the transform


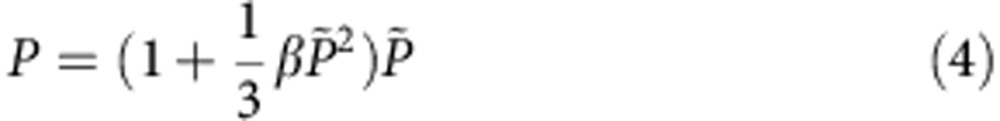


discussed, for example, in ref. 22. As we will see later, to our purpose 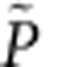 is just an auxiliary operator, we do not need to decide if either *P* or 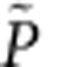 corresponds to the classical momentum. *Q* and 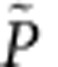 obey the (non deformed) canonical commutation relation 
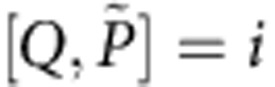
. At the first order in *β*, the Hamiltonian can now be written as


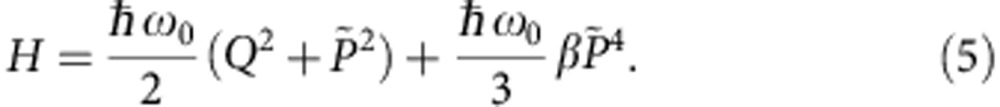


The Heisenberg evolution equations for *Q* and 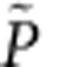, using the Hamiltonian (5) read


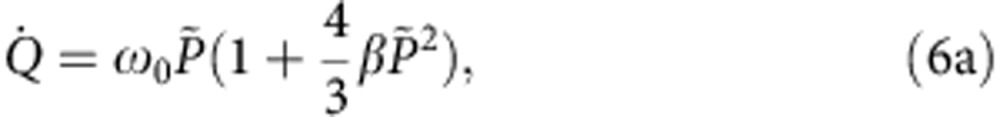



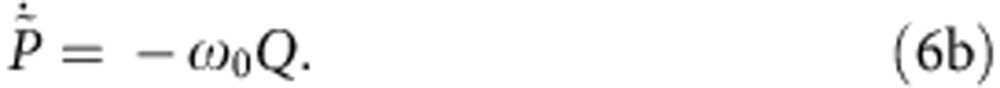


The coupled relations (6) are formally equivalent to the equations describing the evolution of a free anharmonic oscillator with position 
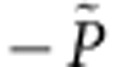
 (*Q* is its conjugate momentum), in a potential 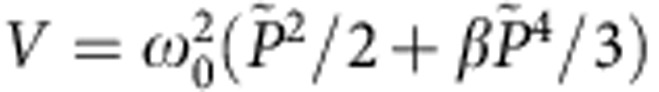 containing a fourth-order component.

The Poincaré's solution^24^, for initial conditions 
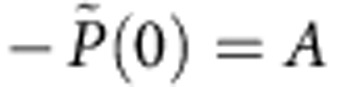
 and 
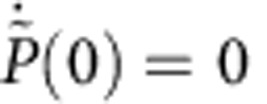
, is 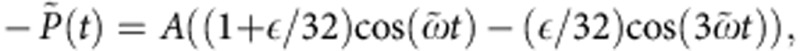 where 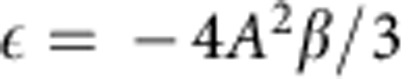 and 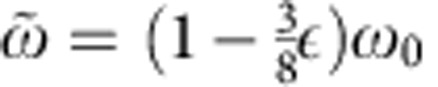. The solution is valid at the first order in 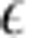, and implies two relevant effects with respect to the harmonic oscillator: the appearance of the third harmonic and, less obvious, a dependence of the oscillation frequency on the amplitude (more precisely, a quadratic dependence of the frequency shift on the oscillation amplitude).

Using again Equation 6b to find *Q*(*t*), keeping the first order in 
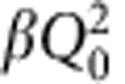
 where *Q*_0_ is the oscillation amplitude for *Q*, we obtain


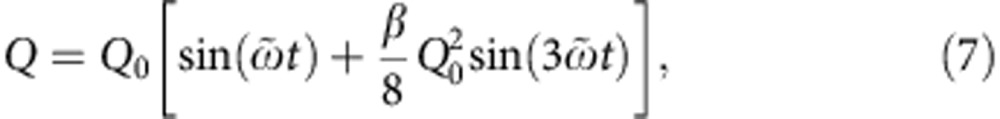


where


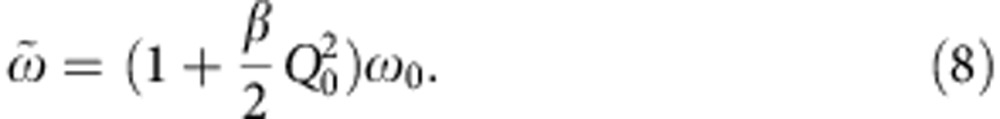


The position *Q*(*t*) is our meaningful (that is, measured) variable, whatever is the physical meaning of *P* and 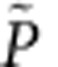. P. Pedram calculates in ref. 19 the evolution of an harmonic oscillator with an Hamiltonian deformed according to the GUP considered in this work, and finds a frequency modified as (in our notation) 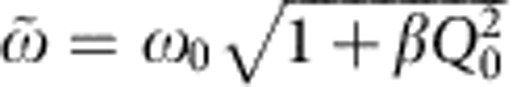. Such expression is equivalent to Equation 8 in the limit of small 
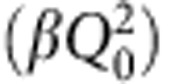
, satisfied in the present work.

We have performed the experiments with highly isolated oscillators, that is, with a high mechanical quality factor *Q*_m_:=*ω*_0_*τ*, where *τ* is a long but nonetheless finite relaxation time, responsible for an additional term −2*P*/*τ* in the right-hand side of Equation 6b. Damping has a twofold effect: (i) an exponential decay of the oscillation amplitude; (ii) a nontrivial time-dependence of the phase. In the limit *Q*_m_≫1, the dynamics is described by a modified version of Equation 7 with the replacements 
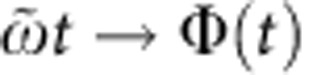
, implying 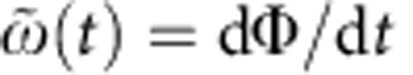 and *Q*_0_→*Q*_0_ exp(−*t*/*τ*). More details on the inclusion of damping in the evolution equations are reported in the Supplementary Notes 1 and 2.

### Experimental apparatus

We have examined three kinds of oscillators, with masses of respectively ≈10^−4^, ≈10^−7^ and ≈10^−11^ kg. The measurements are performed by exciting an oscillation mode and monitoring a possible dependence of the oscillation frequency and shape (that is, harmonic contents) on its amplitude, during the free decay. To keep a more general analysis, we will consider both indicators independently.

**Figure 1:**
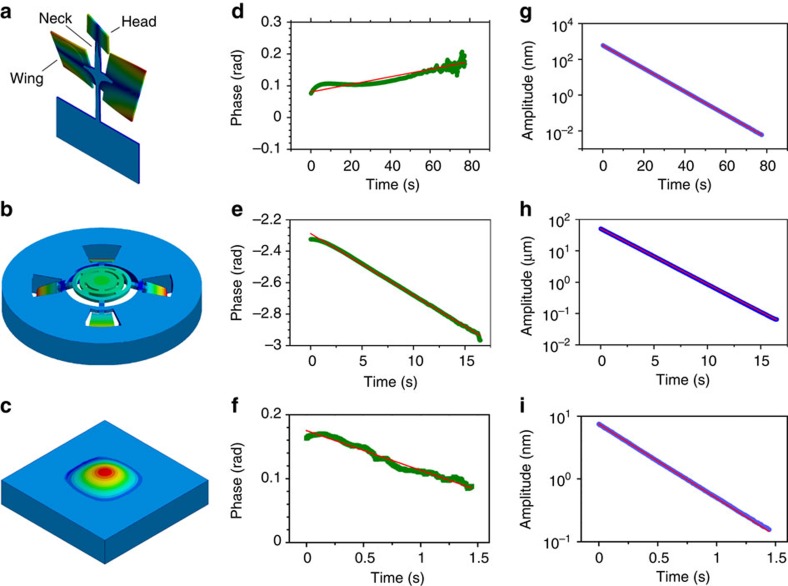
Oscillating devices. Finite elements simulation of the shapes of the oscillation modes investigated in this work (**a**, **b**, **c**), phase (**d**, **e**, **f**) and amplitude (**g**, **h**, **i**) of the oscillation during a free decay, obtained by phase-sensitive analysis of the measured position. In the left panels, the colour scale represents the relative magnitude of the displacement for each modal shape, decreasing from red to blue. Red solid lines: linear and exponential fits respectively to the phase (blue dots) and the amplitude (green dots) experimental data. Graphs (**a**, **d**, **g**) refer to the DPO oscillator, consisting of two inertial members, head and a couple of wings, linked by a torsion rod (the neck) and connected to the outer frame by a leg. The displayed AS mode consist of a twist of the neck around the symmetry axis and a synchronous oscillation of the wings. The elastic energy is primarily located at the neck, where the maximum strain field occurs during the oscillations, while the leg remains at rest and the foot can be supported by the outer frame with negligible energy dissipation. Graphs (**b**, **e**, **h**) refer to the balanced wheel oscillator. The central disk has a diameter of 0.54 mm, and the shape of the beams maintain it flat during the motion (as shown by its homogeneous colour) reducing the dissipation on the 0.4-mm diameter optical coating. The four paddles are carefully sized in order to balance the stress induced by the strain of the beams on the supporting wheel, such that the joints correspond to nodal points. An additional external wheel further improves the isolation from the background. Graphs (**c**, **f**, **i**) refer to the *L*=0.5-mm side, 30-nm thick, square membrane of stoichiometric SiN membrane. Its high stress increases the mechanical quality factor thanks to the dilution effect.

The first device is a ‘double paddle oscillator' (DPO)^25^ made from a 300-μm thick silicon plate (Fig. 1a). Thanks to its shape, for two particular balanced oscillation modes, the antisymmetric torsion modes (AS), the oscillator is supported by the outer frame with negligible energy dissipation and it can therefore be considered as isolated from the background^26^. Vibrations are excited and detected capacitively, thanks to two gold electrodes evaporated over the oscillator, and two external electrodes. The sample is kept in a vacuum chamber, and its temperature is stabilized at 293 K within 2 mK, a crucial feature to maintain a constant resonance frequency during the measurements. We have monitored the AS2 mode, with a resonance frequency of 5636 Hz and a mechanical quality factor of 1.18 × 10^5^ (at room temperature). The overall c.m. of the oscillator remains at rest during the AS motion. To our purpose, we consider the positions of the couple of c.m.'s corresponding to the two half-oscillators that move symmetrically around the oscillator rest plane (a deeper discussion of this issue is reported in ref. 10). The meaningful mass is the reduced mass of the couple of half-oscillators, that is calculated by Finite Element Method simulations and is *m*=0.033 g.

For the measurements at intermediate mass we have used a silicon wheel oscillator, made on the 70-μm thick device layer of an Silicon-On-Insulator wafer and composed of a central disk kept by structured beams^27^, balanced by four counterweights on the beams joints that so become nodal points (Fig. 1b)^28^. On the surface of the central disk, a multilayer SiO_2_/Ta_2_O_5_ dielectric coating forms an high reflectivity mirror. The device also includes intermediate stages of mechanical isolation. The design strategy allows to obtain a balanced oscillating mode (its resonance frequency is 141,797 Hz), with a planar motion of the central mass (significantly reducing the contribution of the optical coating to the structural dissipation) and a strong isolation from the frame. The oscillator is mechanically excited using a piezoelectric ceramic glued on the sample mount. The surface of the core of the device works as end mirror in one arm of a stabilized Michelson interferometer, that allows to measure its displacement. The quality factor surpasses 10^6^ at the temperature of 4.3 K, kept during the measurements. As for the DPO, the c.m. of the oscillator remains at rest and, for the following analysis of the possible quantum gravity effects, we consider the reduced mass *m*=20 μg. We have also performed room temperature measurements on a simpler device, lacking of counterweights, with an oscillating mass of 77 μg and a frequency of 128,965 Hz.

Finally, the lighter oscillators is a *L*=0.5 mm side, 30 nm thick, square membrane of stoichiometric silicon nitride, grown on a 5 mm × 5 mm, 200-μm thick silicon substrate^29^. Thanks to the high tensile stress, the vibration can be described by standard membrane modes, the lowest one (monitored in this work) with shape *z*(*x*,*y*)=*A* cos(*πx*/*L*) cos(*πy*/*L*), where (*x*,*y*) are the coordinates measured from the membrane centre, along directions parallel to its sides (Fig. 1c). The physical mass of the membrane is 20 ng, respectively, and the c.m. is at the position (0,0,*z*_cm_) with *z*_cm_=4*A*/*π*^2^ (the central position *A* is the monitored observable). We have performed the measurements in a cryostat at the temperature of 65 K and pressure of 10^−4^ Pa, where the oscillation frequency is 747 kHz and the quality factor is 8.6 × 10^5^. Excitation and readout are performed as in the experiment with the wheel oscillators.

### Measurements and data analysis

**Figure 2:**
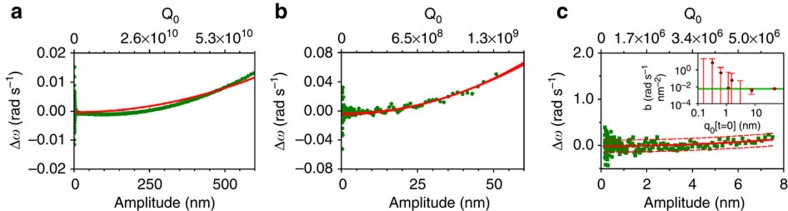
Residual angular frequency fluctuations as a function of the oscillation amplitude. The fluctuations Δ*ω* are measured during the free decay, for the DPO (**a**), wheel (**b**) and membrane (**c**) oscillators. On the upper axes, the same oscillation amplitudes are normalized to the respective oscillator ground state wavefunction width 
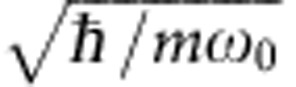
. Red solid lines are the fits with Equation 8, dashed lines report the 95% confidence area. In the inset, we report the values of the quadratic coefficient *b* measured for the membrane oscillator at different excitation amplitudes, with their 95% confidence error bars (for appreciating the improvement in the accuracy, we just show the positive vertical semi-axis in logarithmic scale). For the two points at highest amplitude, the measured *b* is significantly different from zero. The green lines show the interval of *b* calculated from the nonlinear behaviour observed in the frequency domain for stronger excitation.

The first step in the data analysis is applying to the data stream *q*(*t*) a numerical lock-in: the two quadratures *X*(*t*) and *Y*(*t*) are calculated by multiplying the data respectively by sin(*ω*_0_*t*) and cos(*ω*_0_*t*), where *ω*_0_ is the oscillation angular frequency of the acquired time series, estimated preliminarily from a spectrum, and applying appropriate low-pass filtering. The oscillation amplitude is calculated as 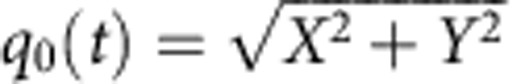 and the phase as Φ(*t*)=arctan(*Y*/*X*). For the DPO oscillator, this process is directly performed by the hardware lock-in amplifier, that is also used to frequency downshift the signal of the wheel oscillator at cryogenic temperature before its acquisition. *q*_0_(*t*) is fitted with an exponential decay (examples are shown in Fig. 1), while Φ(*t*), that always remains within ±*π* rad, is fitted with a linear function that gives the optimal frequency and phase with respect to the preliminary tries *ω*_0_ and Φ(0)=0. The residuals ΔΦ of the fit are differentiated to estimate the fluctuations Δ*ω* in the oscillation frequency. In Fig. 2, we show Δ*ω* as a function of *q*_0_, together with its fit with the function 
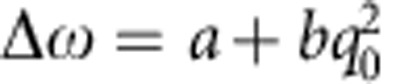
. The derived value and uncertainty in the quadratic coefficient *b* are the meaningful quantities that can be used to establish upper limits to the deformation parameter *β*_0_. The background mechanical noise in all the experiments is dominated by the oscillator thermal noise (as verified with spectra taken without excitation). The consequent statistical uncertainty in the calculated Δ*ω* is inversely proportional to the amplitude *q*_0_, and such a weight is indeed used in the fitting procedures.

In the case of the DPO oscillator (Fig. 2a), Δ*ω* versus *q*_0_ has a clear shape that is given by the intrinsic oscillator nonlinearity. A similar, weaker effect is observed for the wheel oscillator at cryogenic temperature and for the membrane, at the largest excitation amplitudes (Figs 2b,c). The quadratic coefficient for the membrane is in agreement with its calculation based on the nonlinear behaviour observed for larger amplitudes in the frequency domain^30^. Since structural nonlinearity is hard to model, we cannot distinguish it from possible quantum gravity effects. Therefore, we just place our upper bound in correspondence of the first (the strongest) nonlinear behaviour encountered. The meaningful quantity to calculate an upper limit to *β*_0_ is the mean value of *b* plus its uncertainty. The latter is calculated from the standard deviation on several independent measurements, and it is in agreement with the error estimated from the residuals of each fit, after decimation of the data sets to obtain uncorrelated data points. The experiment has been repeated for several excitation levels, finding the expected improvements in the upper limit to *b* at increasing amplitudes (inset of Fig. 2c).

As previously discussed, a further useful indicator is the amplitude of the third harmonic component, also extracted with a lock-in procedure. The value of *β* inferred from such parameter using Equation 7, is to ascribe to the relatively poor linearity of the readout, and is therefore considered as an upper limit to possible quantum gravity effects.

A model-independent constraint to possible effects of a deformed commutator can be derived from the residual frequency fluctuations Δ*ω*, considered as a function of the oscillation amplitude (reported in Fig. 2). To this purpose, we summarize in Table 1 the maximum relative frequency shift and the maximum dimensionless oscillation amplitude *Q*_0_(0), for the different oscillator masses examined in this work. These data can be used to test any modified dynamics and provide the consequent upper limits to the involved parameters.

**Figure 3:**
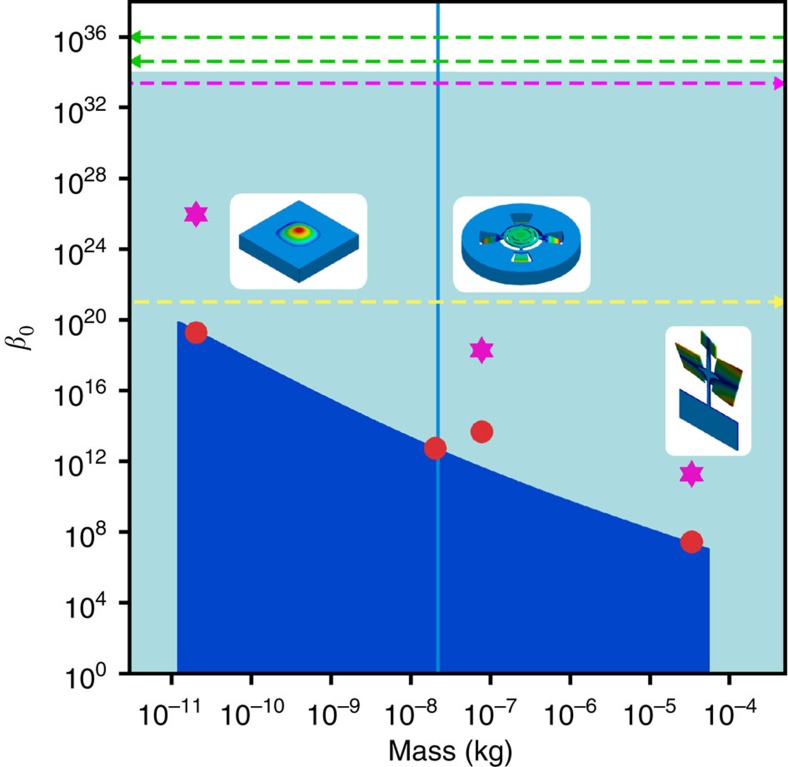
Upper limits to the deformed commutator. The parameter *β*_0_ quantifies the deformation to the standard commutator between position and momentum, or the scale 
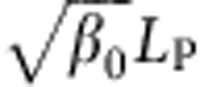
 below which new physics could come into play. Full symbols reports its upper limits obtained in this work, as a function of the mass. Red dots: from the dependence of the oscillation frequency from its amplitude; magenta stars: from the third harmonic distortion. In the former data set, for the intermediate mass range (10–100 μg), we report the results obtained with two different oscillators. Light blue shows the area below the electroweak scale, dark blue the area that remains unexplored. Dashed lines report some previously estimated upper limits, obtained in mass ranges outside this graph (as indicated by the arrows). Green: from high-resolution spectroscopy on the hydrogen atom, considering the ground state Lamb shift (upper line)^21^ and the 1S–2S level difference (lower line)^22^. Magenta: from the AURIGA detector^10^,^11^. Yellow: from the lack of violation of the equivalence principle^39^. The vertical line corresponds to the Planck mass (22 μg).

For a more accurate and specific bound, we focus on the model described by Equations 7 and 8. The values and uncertainties in *β* and *β*_0_ are obtained from *b* and from the third harmonic distortion, using the oscillator parameters (namely, its mass and frequency). In Table 1 we summarize our results for the different upper limits, given at the 95% confidence level. The results for *β*_0_ are also displayed in Fig. 3 as a function of the oscillator mass, and compared with some previously existing limits. We have achieved a significant improvement, by many orders of magnitude, working on systems with disparate mass scales and considering different measured observables.

## Discussion

We have performed an extended experimental analysis of the possible dependence of the oscillation frequency and third harmonic distortion on the oscillation amplitude in micro- and nano-oscillators, spanning a wide range of masses. Assuming that a deformed commutator between position and momentum governs the dynamics through standard Heisenberg equations, we obtain a reduction by many orders of magnitude of the previous upper limits to the parameters quantifying the commutator deformation. We remark that the measurements have been performed on state of the art oscillators, allowing low statistical uncertainty (because of the high mechanical quality factor), low background noise (thanks to the shot-noise limited detection and the cryogenic environment), high-frequency stability (beyond the resonance linewidth), and the highest excitation amplitude allowed by each oscillator. The latter condition is not commonly explored in metrological micro- and nano-oscillators^31^,^32^, and we could indeed achieve the limit given by the intrinsic oscillators nonlinearity. These effects are not well mastered at present, therefore we have kept the conservative attitude of setting an upper limit to the overall nonlinear behaviour, which includes possible quantum gravity effects. A detailed modelling of the structural nonlinearity could allow in the future to subtract their effects from our data (in particular in the case of the DPO, for which the shape of Δ*ω* versus *q*_0_ is clearly different from a parabola), and thus set even stronger limits to the remaining nonlinearity and actually to *β*_0_. The mentioned crucial properties (high *Q*_m_, high-resonance frequency at a given mass, high-frequency stability) must be conserved or improved in possible further experiments aiming to lower the bounds on the deformation parameters. We stress however that, at present, the wall in our experiment is given by dynamic range, actually determined by the structural nonlinearity that should be reduced to improve the results. In this regard, an interesting possibility to be explored is the use of high-quality bulk crystalline resonators^32^,^33^.

Extending the use of the Heisenberg evolution equations with deformed commutators from an ideal particle to a macroscopic dynamics is not free from conceptual problems^34^.

A direct extrapolation from quantum to classical dynamics, discussed, for example, in refs 35, 36, 37, implies crucial consequences, the first being the violation of the equivalence principle^38^,^39^,^40^. Current bounds to such violation, obtained using sensitive torsion balances^41^, correspond to a deformation parameter *β*_0_<10^21^(ref. 39). Our limits are substantially lower. Our model shows that remarkable deviations from classical trajectories are, in any case, expected as soon as the momentum is of the order of (or exceeds) *m*_P_*c*. This condition is straightforward at astronomic level^42^, and even for kilogram-scale bodies. This may either indicate the breakdown of the Eherenfest' theorem at all scales^18^ (requiring to revise the rules connecting quantum to classical dynamics), or a possible mass dependence of the deformation parameter. In this context our experiments, involving a wide range of masses, taking as ‘natural' reference the Planck mass, become particularly meaningful. Our experimental results, when used to set limits on the deformed commutator described in Equation (2), should not be simply intended as a check of possible deformations of quantum mechanics, but as a test of a ‘composite' hypothesis, involving also the form of the classical limit corresponding to the modified quantum rules.

As a second general remark, it should be underlined that the role of the c.m. coordinates in a deformed space is still a matter of debate. As recently remarked in ref. 43, the motion of the c.m. typically do not involve a Planck energy concentrated in a Planck-scale volume. In the same article, the author constructs a deformed commutator for a composite system starting from a number *N* of elementary constituents, and shows that the deformation parameter should scale as *N*^−2^. Therefore, even assuming the constituent particles to be affected by Planck scale physics, the c.m. of a composite macroscopic body would be much more weakly affected. This approach leads to the interesting (maybe troubling) conclusion that free elementary particles should feel quantum gravitational effects in a different way with respect to, for example, protons or atoms or, in other words, that spacetime properties should depend on the kind of particle^22^,^44^. We further remark that, at the present stage, we do not know at which constituent-particle level quantum gravity effects could intervene^44^, and there are no theories even suggesting what such ‘elementary constituents' should be. Other works suggest instead that the effects should scale as the number of elementary interactions^45^.

A different point of view is to consider the effects of an intrinsically discrete spacetime on the dynamics of a quantum system. We remark that, in quantum mechanics, the wavefunction associated to the c.m. has properties that cannot be simply reduced to the coordinates of the constituent particles. It has been shown that a discretization of spacetime, (for example, related to the creation and annihilation of particle–antiparticle pairs) would naturally suggest discretization of the Hilbert space associated to the considered quantum system^46^. Although our universe might still be infinite in extent, any experiment or observation involves just a finite region of spacetime. In ref. 47 is investigated the emergence of extended uncertainty relations for discrete coordinate and momentum operators in such finite discrete configuration spaces, which can be formulated in the form of a GUP. In this context, deformed commutators appear as the manifestation of the background discreteness, with the minimal scale being a fundamental property of spacetime. In this case, one could expect also the low-energy motion of a macroscopic body to be affected, independently on the measurement process.

Although all these approaches are formally correct, they rely on very different hypothesis, whose validity should be checked by experimental measurements. This represents a strong motivation for the realization of experiments involving macroscopic mechanical oscillators. We notice that clear quantum signatures have been recently obtained even in ‘macroscopic' nano-oscillators^48^,^49^,^50^,^51^ very similar to those exploited in this work, suggesting that well isolated mechanical oscillators are indeed privileged experimental systems to explore the classical-to-quantum transition. Since gravity effects could have a role in the wavefunction decoherence that marks such transition^52^,^53^, and it cannot be excluded that quantum gravity is inextricably linked to peculiar quantum features, an intriguing extension of the present experiment (or a similar investigation) would naturally be performed with macroscopic oscillators in a fully quantum regime.

## Additional information

**How to cite this article**: Bawaj, M. *et al.* Probing deformed commutators with macroscopic harmonic oscillators. *Nat. Commun.* 6:7503 doi: 10.1038/ncomms8503 (2015).

## Supplementary Material

Supplementary Information Supplementary Notes 1-2 and Supplementary References

## Figures and Tables

**Table 1:** Results of the experiment.

**Mass (kg)**	**Frequency (Hz)**	**Max. ampl. (nm)**	**Max.** ***Q***_**0**_	**Max. Δ*****ω*****/*****ω***_**0**_	***β***	***β***_**0**_	**Indicator**
3.3 × 10^−5^	5.64 × 10^3^	600	6 × 10^10^	4 × 10^−7^	7 × 10^−29^	3 × 10^7^	Δ*ω*
3.3 × 10^−5^	5.64 × 10^3^				7 × 10^−25^	2 × 10^11^	Third harmonic
7.7 × 10^−8^	1.29 × 10^5^				8 × 10^−24^	5 × 10^13^	Δ*ω*
7.7 × 10^−8^	1.29 × 10^5^				2 × 10^−19^	2 × 10^18^	Third harmonic
2 × 10^−8^	1.42 × 10^5^	55	7 × 10^8^	6 × 10^−8^	3 × 10^−25^	6 × 10^12^	Δ*ω*
2 × 10^−11^	7.47 × 10^5^	7.5	7 × 10^6^	4 × 10^−8^	4 × 10^−21^	2 × 10^19^	Δ*ω*
2 × 10^−11^	7.47 × 10^5^	47	4 × 10^7^	3 × 10^−6^	4 × 10^−21^	2 × 10^19^	Δ*ω*
2 × 10^−11^	7.47 × 10^5^				2 × 10^−14^	1 × 10^26^	Third harmonic

Maximum relative frequency shifts measured for different oscillators, corresponding oscillation amplitudes, and upper limits to the deformation parameters *β* and *β*_0_ obtained in this work.
