# Supplementary material for: Probing deformed commutators with macroscopic harmonic oscillators
Source: Nat Commun. 2015 Jun 19;6:7503. doi: 10.1038/ncomms8503 (PMC4557370; doi:10.1038/ncomms8503)
Supplement: Supplementary Information — Supplementary Notes 1-2 and Supplementary References [file ncomms8503-s1.pdf]

Supplementary note 1: adding damping to the evolution in the presence of deformed commutators

Our analysis is based on two basic simple and reasonable assumptions: i) the time evolution of a system is generated by its Hamiltonian, and therefore it is dictated by the Heisenberg equation; ii) the Hamiltonian of an harmonic oscillator with position q and conjugated momentum p is the usual one, $H = m\omega_0^2 q^2/2 + p^2/2m$. The first assumption is motivated by the connection between time translations and a system Hamiltonian, while the second assumption is motivated by an overwhelming set of experimental results. Therefore the only effect of the existence of a minimal length is associated with a deformation of the commutator between position and momentum of the harmonic oscillator,

$$[q, p] = i\hbar\left[1 + \beta_0\left(\frac{p}{m_{\mathrm{P}}c}\right)^2\right], \tag{1}$$

where m_{P} is the Planck mass ($\approx 22\,\mu$g), and β_0 is a dimensionless parameter that we are limiting with the experiment.

We first define the usual dimensionless coordinates Q and P, according to $q = \sqrt{\hbar/(m\omega_0)}\,Q$ and $p = \sqrt{\hbar m\omega_0}\,P$. The Hamiltonian is now written in the standard form

$$H_{\mathrm{S}} = \frac{\hbar\omega_0}{2}(Q^2 + P^2) \tag{2}$$

and the commutator in Eq. (1) becomes

$$[Q, P] = i(1 + \beta P^2), \tag{3}$$

where $\beta = \beta_0\,(\hbar m\omega_0/m_{\mathrm{P}}^2c^2)$ is a further dimensionless parameter that we assume to be small ($\beta \ll 1$). Such assumption will have to be consistent with the experimental results.

Damping and noise acting on the resonator are due to the coupling to an external environment which, as extensively discussed in the literature [1, 2, 3, 4], can be modeled in terms of a set of independent harmonic oscillators, with frequency ω_j, couplings k_j, and whose canonical coordinates q_j and p_j have been again rescaled as we have done with the canonical variables of the resonator of interest. The total system Hamiltonian is [1, 2, 3]

$$H_{\mathrm{S}} + \sum_j \frac{\hbar\omega_j}{2}\left[p_j^2 + \left(q_j - k_j Q\right)^2\right], \tag{4}$$

and the quantum Langevin equations for the operators Q and P can be obtained starting from the Heisenberg equations for $q(t)$, $p(t)$ associated with this Hamiltonian. We assume the usual commutation rules for the reservoir oscillators $[q_j, p_k] = i\delta_{jk}$, i.e., that they are *not* modified by minimum length arguments, and we will justify this choice later on.

The corresponding evolution equations are

$$\dot{Q}(t) = \frac{i}{\hbar}[H_{\mathrm{S}}, Q(t)] \tag{5}$$

$$\dot{P}(t) = \frac{i}{\hbar}[H_{\mathrm{S}}, P(t)] + \sum_j \omega_j k_j \left[q_j(t) - k_j Q(t)\right] \tag{6}$$

$$\ddot{q}_j(t) = -\omega_j^2\left[q_j(t) - k_j Q(t)\right]\;. \tag{7}$$

We now proceed in the usual way by formally solving the dynamics of the reservoir oscillators $q_j(t)$,

$$q_j(t) = q_j(t)^{(0)} + k_j \int_0^t ds\,\omega_j \sin\omega_j(t-s)Q(s), \tag{8}$$

where $q_j(t)^{(0)} = q_j(0)\cos(\omega_j t) + p_j(0)\sin(\omega_j t)$ is the free dynamics of each oscillator. We now integrate by parts the integral on the right hand side and rewrite Eq. (8) as

$$q_j(t) - k_j Q(t) = q_j(t)^{(0)} - k_j\cos(\omega_j t)\,Q(0) - \int_0^t ds\,k_j\cos\omega_j(t-s)\dot{Q}(s). \tag{9}$$

We now insert this formal solution into Eq. (6), so that we finally get the general Langevin equations for the resonator of interest,

$$\dot{Q}(t) = \frac{i}{\hbar}[H_{\mathrm{S}}, Q(t)] \tag{10}$$

$$\dot{P}(t) = \frac{i}{\hbar}[H_{\mathrm{S}}, P(t)] - K(t)Q(0) - \int_0^t ds\,K(t-s)\dot{Q}(s) + F(t), \tag{11}$$

where $K(t) = \sum_j \omega_j k_j^2 \cos(\omega_j t)$ is the reservoir memory kernel function and $F(t) = \sum_j k_j \omega_j q_j(t)^{(0)}$ is a reservoir operator which must be interpreted as a thermal stochastic force operator with zero mean value.

As it is well known, irreversible damping due to the reservoir is obtained only when an infinite number of oscillators, distributed over a continuum of frequencies, is considered. The simplest option is to perform the continuous limit according to the usual Markovian prescription of a flat spectral distribution,

$$\sum_j \omega_j k_j^2 \cdots \to \int_0^{+\infty} d\omega\, \omega k^2(\omega) \frac{dn}{d\omega} \cdots = \frac{\gamma}{\omega_0 \pi} \int_0^{+\infty} d\omega \cdots , \tag{12}$$

where $dn/d\omega$ is the oscillators density, and γ is just the damping rate of the resonator, which is associated with the overall coupling strength between the oscillator and its reservoir. With this choice, we have $K(t) = (\gamma/\omega_0)\delta(t)$, where $\delta(t)$ is the Dirac delta function, so that the Heisenberg equations for the oscillator of interest become

$$\dot{Q}(t) = \frac{i}{\hbar}[H_S, Q(t)] \tag{13}$$

$$\dot{P}(t) = \frac{i}{\hbar}[H_S, P(t)] - \frac{\gamma}{\omega_0}\dot{Q}(t) + F(t)\ . \tag{14}$$

We can now justify why we have assumed unmodified commutators for the reservoir oscillators. These oscillators are fictitious systems describing unspecified excitations of the reservoir, affecting the dynamics of our system of interest only through the spectral density of the coupling coefficients, determining the damping force acting on it. Therefore, it is not evident at all that modified commutators must be considered for such fictitious objects, and even when assuming modified commutators, their overall effect on the reservoir spectral density and on the damping rate γ would be typically negligible.

We now introduce the auxiliary operator $\tilde{P}$ related to the momentum P by the equation

$$P = \left(1 + \frac{1}{3}\beta \tilde{P}^2\right)\tilde{P} \tag{15}$$

which, as shown in Ref. [5], is such that Q and $\tilde{P}$ obey the usual (non deformed) canonical commutation relation $[Q, \tilde{P}] = i$ at first order in β. Therefore, at first order in β, the equation of motion for Q and $\tilde{P}$ become

$$\dot{Q} = \omega_0 \tilde{P}\left(1 + \frac{4}{3}\beta \tilde{P}^2\right), \tag{16a}$$

$$\dot{\tilde{P}} = -\omega_0 Q - \frac{\gamma}{\omega_0}\dot{Q}\left(1 - \beta \tilde{P}^2\right), \tag{16b}$$

where we have also neglected the thermal noise term because we will consider the evolution of mean values from now on. At the first order in β, the damping term in Eq. (16b) can be written as $-2P/\tau$. By taking the derivative of Eq. (16b) and using Eq. (16a), we get the following equation for the auxiliary variable $\tilde{P}$

$$\ddot{\tilde{P}} + \gamma \dot{\tilde{P}}\left(1 + \beta \tilde{P}^2\right) + \omega_0^2 \tilde{P} + \frac{4}{3}\beta \omega_0^2 \tilde{P}^3 = 0. \tag{17}$$

The physical quantity of interest, which is the one really measured in our experiments, is however $Q(t)$, which is obtained from the knowledge of $\tilde{P}(t)$ stemming from the solution of Eq. (17), by formally solving Eq. (16a) for $Q(t)$ which, assuming the initial condition $Q(0) = 0$, gives

$$Q(t) = \omega_0 \int_0^t ds\, \tilde{P}(s)\left(1 + \frac{4}{3}\beta \tilde{P}^2(s)\right). \tag{18}$$

Supplementary note 2: approximate solution of the evolution equation

We have now to solve Eq. (17) at first order in β and also exploiting the weak damping condition $\mathcal{Q}_m \gg 1$. We adopt a multiple scale approach in which the small nonlinear terms at first order in β introduce two first order modifications to the damped oscillatory solution at zeroth order in β of Eq. (17): i) a slowly varying amplitude of the zeroth order solution; ii) addition of a small third harmonic component. The trial solution is therefore

$$\tilde{P}(t) = A_1(t)e^{\lambda t} + A_3(t)e^{3\lambda t} + \text{c.c}, \tag{19}$$

where $\lambda = -\gamma/2 + i\omega_1$, with $\omega_1 = \sqrt{\omega_0^2 - \gamma^2/4}$, is the complex eigenvalue associated with the zeroth-order linear equation, and $A_3(t)$ is at first order in β. Separating the terms with damped oscillations at the frequency ω_1 from those oscillating at $3\omega_1$, we get the following two equations for $A_1(t)$ and $A_3(t)$ (again at first order in β):

$$\ddot{A}_1(t) + i2\omega_1\dot{A}_1(t) + 4\beta\omega_0^2 A_1(t)|A_1(t)|^2 e^{-\gamma t} + \gamma\beta\left[2|A_1(t)|^2\left(\dot{A}_1(t) + \lambda A_1(t)\right) + A_1(t)^2\left(\dot{A}_1^*(t) + \lambda^* A_1^*(t)\right)\right]e^{-\gamma t} = 0, \quad (20)$$

$$\ddot{A}_3(t) + (6\lambda + \gamma)\dot{A}_3(t) + \left(9\lambda^2 + 3\lambda\gamma + \omega_0^2\right)A_3(t) + \frac{4}{3}\beta\omega_0^2 A_1^3(t) + \gamma\beta\left(\dot{A}_1(t) + \lambda A_1(t)\right)A_1(t)^2 = 0. \quad (21)$$

One has to solve Eq. (20) for $A_1(t)$ and then insert this solution into Eq. (21) in order to get the solution for $A_3(t)$. Since $A_1(t)$ is slowly varying we can neglect the second order derivative $\ddot{A}_1(t)$ in Eq. (20); moreover for typical values of the mechanical quality factor, the last term on the left hand side of Eq. (20), is much smaller that the others. Under these two assumptions, which are well justified in the parameter regime of our experiments, Eq. (20) can be easily solved because $|A_1(t)|^2$ is a constant of motion, and one gets

$$A_1(t) = A_1(0)\exp\left[2i\frac{\beta\omega_0^2}{\omega_1\gamma}\left(1 - e^{-\gamma t}\right)|A_1(0)|^2\right]. \quad (22)$$

For what concerns the solution for $A_3(t)$ we notice that Eq. (21) is an inhomogeneous second order linear differential equation for $A_3(t)$ with constant coefficients, in which the last two terms in the left hand side depending upon $A_1(t)$ act as driving terms. Therefore the solution is the sum of two components: i) the general solution of the associated homogeneous equation; ii) a particular solution of the inhomogeneous equation. However the first component, when multiplied by $e^{3\lambda t}$ will only provide a small correction to the term proportional to $e^{\lambda t}$, i.e., a negligible correction to $A_1(t)$, and therefore the relevant solution is only the second component. At first order in β such particular solution is the slowly varying solution obtained by neglecting both $\ddot{A}_3(t)$ and $\dot{A}_3(t)$. Neglecting again the last term on the left hand side of Eq. (21) proportional to $\gamma\beta$ due to large mechanical quality factor , we fin ally get

$$A_3(t) = -\frac{2\beta\omega_0^2}{3\lambda(4\lambda + \gamma)}A_1^3(t) \quad \simeq \frac{\beta}{6}A_1^3(t) \ \text{ in the limit of high } Q_m. \quad (23)$$

In Eq. (23) we have used the fact that $\omega_0^2 = -\lambda^2 - \gamma\lambda$ in the denominator. If we now insert Eqs. (22)-(23) into the trial solution of Eq. (19) and the resulting expression into Eq. (18), at the first order in β the position $Q(t)$ can be written as

$$Q(t) = \omega_0 \int_0^t ds\, A_1(s)e^{\lambda s} + \left(A_3(s) + \frac{4}{3}\beta A_1^3(s)\right)e^{3\lambda s} + \text{c.c} \quad \simeq \omega_0 \int_0^t ds\, A_1(s)e^{\lambda s} + \frac{3}{2}\beta A_1^3(s)\, e^{3\lambda s} + \text{c.c.}\,. \quad (24)$$

By taking $A_1(s)$ and $A_1^3(s)$ out of the integral as slowing varying envelops and performing the integration on $e^{\lambda s}$ and $e^{3\lambda s}$, defining $Q_0 = 2A_1(0)$ and $\tau = 2/\gamma$ (amplitude decay time), we obtain

$$Q(t) = \frac{\omega_0}{2\lambda}Q_0\, e^{-\frac{t}{\tau}}\left[e^{i\Phi(t)} + \frac{\beta}{8}Q_0^2\, e^{-2\frac{t}{\tau}}e^{i3\Phi(t)}\right] + \text{c.c.} \quad (25)$$

where the time-dependent phase $\Phi(t)$ is

$$\Phi(t) = \frac{\beta}{2}\frac{\omega_0^2}{\omega_1}\frac{1 - e^{-\gamma t}}{\gamma}Q_0^2 + \omega_1 t = \int_0^t ds\, \omega_1\left[1 + \frac{\beta}{2}\frac{\omega_0^2}{\omega_1^2}Q_0^2\, e^{-2\frac{s}{\tau}}\right] \quad (26)$$

and, again in the limit of high Q_m (i.e., $\omega_0/\omega_1 \simeq 1$ and $\omega_0/\lambda \simeq 1/i$), we find the expression described in the main text.

We have performed a numerical integration of Eqs. (16) with our typical experimental parameters, and we have indeed found that our final expression of $Q(t)$, used for the analysis of the experimental data, well reproduces the simulation.

Supplementary References

[1] Caldeira, A. O. & Leggett, A. J. Quantum tunnelling in a dissipative system. *Ann. Phys. (N.Y.)* **149**, 374 (1983).
[2] Gardiner, C. W. & Zoller, P. *Quantum Noise* (Springer-Verlag, Berlin, 2004), Chap. 3.
[3] Ford, G. W., Lewis, J. T. & O'Connell, R. F. Quantum Langevin equation. *Phys. Rev. A* **37**, 4419 (1988).
[4] Giovannetti, V., Vitali, D. Phase-noise measurement in a cavity with a movable mirror undergoing quantum Brownian motion. *Phys. Rev. A* **63**, 023812 (2001).
[5] Quesne, C. & Tkachuk, V. M. Composite system in deformed space with minimal length. *Phys. Rev. A* **81**, 012106 (2010).
